# Psoriatic arthritis

**DOI:** 10.12688/f1000research.19144.1

**Published:** 2019-09-20

**Authors:** Vanessa Ocampo D, Dafna Gladman

**Affiliations:** 1 University of Toronto, Psoriatic Arthritis Program, University Health Network, Toronto Western Hospital, Toronto, Ontario, Canada; 2 Krembil Research Institute, Toronto Western Hospital, Toronto, Ontario, Canada

**Keywords:** psoriatic arthritis, pathogenesis, treatment, biomarkers

## Abstract

Psoriasis is a multisystemic, inflammatory skin condition that can affect many areas of the body, but most commonly the extensor surfaces of the elbows and knees, and sometimes the intergluteal and umbilical area. It has a prevalence of 2–4% in western adults, and 20­–30% of psoriasis patients will develop psoriatic arthritis (PsA). PsA is an inflammatory musculoskeletal disease associated with cutaneous psoriasis. It affects men and women almost equally with a peak age at onset of 40 and 50 years. It is a diverse disease that affects multiple organ systems includes peripheral and axial joints, entheses, skin, and nails. PsA is associated with comorbidities such as osteoporosis, uveitis, subclinical bowel inflammation, and cardiovascular disease. Given this heterogeneity, its diagnosis has been difficult. Here we present an updated review of its classification criteria CASPAR (classification criteria for PsA), use of screening tools to aid in early diagnosis, recent findings on pathogenesis, and new therapeutic approaches including new biologic medications.

## Introduction

Psoriasis is a multisystemic, chronic inflammatory skin condition manifesting with scaly erythematous plaques most commonly affecting extensor surfaces of the elbows and knees, and sometimes the intergluteal and umbilical area ^1^, and other parts of the body ^1^. It has a prevalence of 2–4% in Western adults ^2^, and 20–30% of psoriasis patients will develop psoriatic arthritis (PsA) ^3^. In a prospective study of 464 patients with psoriasis who were confirmed not to have inflammatory arthritis at presentation to the clinic, 51 developed PsA during an 8-year follow-up, for an annual incidence of 2.7% ^4^.

PsA is an inflammatory musculoskeletal disease associated with cutaneous psoriasis. It affects men and women almost equally between the ages of 40 and 50 years ^5^. The diversity of affected organ systems includes peripheral and axial joints, entheses, skin, and nails. PsA is associated with comorbidities such as osteoporosis, uveitis, subclinical bowel inflammation, and cardiovascular disease ^6, 7^. Given this heterogeneity, its diagnosis has been difficult. However, classification criteria such as CASPAR (classification criteria for PsA) ^8^ and several screening tools have facilitated the recognition of this disease among family physicians, dermatologists, and rheumatologists.

Up until 20 years ago, there were not many very effective treatments for PsA. Thankfully, over the last decade, there has been a revolution of new therapies with biologics, leading to better control of the disease and its consequent complications and comorbidities. We now know that early diagnosis is important if we are to prevent damage in patients with PsA. This article reviews PsA early diagnosis, recent findings in pathogenesis, and new therapeutic approaches.

## Early diagnosis in psoriatic arthritis

Delayed PsA diagnosis has been shown to be associated with worse physical function ^9^. Haroon *et al*. demonstrated that even a 6-month delay from symptom onset to the first visit to the rheumatologist (delay in consultation) resulted in worse outcome for patients with PsA, with more peripheral joint erosions, sacroiliitis, and worse health assessment questionnaire (HAQ) scores ^10^. This population of patients has increased frequency of cardiovascular disease, diabetes, metabolic syndrome, and depression, which in some cases is triggered by the persistent inflammatory state in untreated PsA ^11^. But how can we diagnose PsA earlier?

### Clinical features

Clinical features of the disease may help to identify patients with psoriasis at risk of developing arthritis. Psoriasis severity increases the risk for PsA, as shown in a large cohort study ^12^. Having more than three body sites affected by psoriasis (compared to one site) was associated with a 2.24-fold increased risk of PsA ^13^. The location of psoriasis can increase the risk of PsA; one study found a 3.98-fold increase in PsA with scalp lesions and another identified a 2.35-fold increase with intergluteal and perianal lesions ^13^. Conversely, as scalp and intergluteal lesions are so often observed in psoriasis, they may not reliably indicate which patients should be referred to a rheumatologist. Nail lesions occur in over 80% of patients with PsA compared to about 40% of patients with psoriasis without arthritis ^14, 15^. A prospective study of 464 patients with psoriasis who were confirmed not to have inflammatory arthritis at presentation to the clinic found that 51 developed PsA during an 8-year follow-up. Baseline variables found as risk factors for the development of PsA were severe psoriasis, low level of education, and the use of retinoids. In a time-dependent analysis, nail pitting and uveitis remained significant in a multivariate model ^4^.

### Classification criteria

Even though the CASPAR criteria were established in patients with long-standing disease, studies have shown that they work well in patients with early disease ^16, 17^. Only rheumatologists can accurately make a diagnosis using these criteria, and that is why screening tools were created.

### Screening tools

The development of screening tools has aided the early detection of PsA. The most frequently used are the Toronto PsA Screen (ToPAS), the Psoriasis Arthritis Screening and Evaluation Questionnaire (PASE), and the Psoriasis Epidemiology Screening Tool (PEST). Despite these screening tools demonstrating good sensitivity and specificity during their development, they did not function very well in the real-world setting ^18, 19^. A study from 2015 suggested that adding axial involvement to the ToPAS (ToPAS 2) questionnaire increases the sensitivity of the test as a screening tool, as axial disease is more likely to be part of the PsA spectrum ^20^. Other tools are the early psoriatic arthritis screening questionnaire (EARP), which was validated in 2012 ^21^; the Screening Tool for Rheumatologic Investigation in Psoriatic Arthritis (STRIPP), which was developed by an Italian group of investigators ^22^ and showed good sensitivity and specificity but needs additional validation; and the Simple Psoriatic Arthritis Screening questionnaire (SiPAS), which was validated by the Italian group led by Salaffi, who demonstrated that having >3 out of 5 questions answered “yes” showed a sensitivity of 79% and specificity of 87% (+likelihood ratio [LR] 6.14) ^23^. See Table 1 for a summary of the screening tools.

**Table 1.** Screening questionnaires for psoriatic arthritis.

SCREENING TOOLS	DESCRIPTION	SENSITIVITY/SPECIFICITY
ToPAS * ^18, 19^	Self-administered 11 questions + pictures and diagrams Max score: N/A	Sensitivity: 70–86.8% Specificity: 93.1%
PASE * ^18^	Self-administered 15 questions Max score: 75	Sensitivity: 59–82% Specificity: 66–73%
ToPAs 2 ^21– 82^		Sensitivity: 44% Specificity: 97%
EARP ^21^	Self-administered 9 questions Max score: 10	Sensitivity: 85% Specificity: 78–85%
STRIPP ^22^	Self-administered	Sensitivity: 91.5% Specificity: 93.3%
SiPAS ^23^	Self-administered 5 questions Max score: 5	Sensitivity: 79% Specificity: 87%
PEST * ^83^	Self-administered 5 questions + joint diagram Max score: N/A	Sensitivity: 68–97% Specificity: 71–79%

*Table adapted from article by Machado *et al*. ^84^

### Sonography

Sonography has a role in identifying early PsA patients ^5^, mostly those who do not have the “classic” initial clinical picture. Gisondi *et al*. used the Glasgow Ultrasound Enthesis Scoring System (GUESS) to compare the enthesis of patients with psoriasis versus controls. They found that the mean GUESS score was higher and the entheses were thicker in psoriasis patients compared to controls. In a 2-year follow up, they found that 3 out of the 30 patients developed PsA ^24^.

In 2019, the GRAPPA ultrasound working group assessed the performance of various sonographic elemental entheseal lesions in distinguishing between PsA and controls, with the aim of informing the development of a novel sonographic enthesitis score for PsA ^25^. They found that the best model, which gave an area under the curve of 0.93, included lesions such as enthesophytes, Doppler signal, erosions, thickening, and hypoechogenicity and six entheseal sites (patellar ligament insertions into the distal patella and tibial tuberosity, Achilles tendon and plantar fascia insertions into the calcaneus, common extensor tendon insertion into lateral epicondyle, and supraspinatus insertion into the superior facet of the humerus ^24^).

### Biomarkers

A biological marker is any component identified via genomic transcription, proteomic, cellular, or imaging approaches that is associated with the pathophysiology, clinical course, or outcome of a specific disease ^26^. For clinicians (especially family doctors, dermatologists, and rheumatologists), having a biomarker would facilitate the identification of individuals likely to develop PsA.

***Genomic biomarkers.*** The HLA alleles that distinguish patients with PsA from those with psoriasis without arthritis have been identified and replicated. The heterogenicity between PsA and psoriasis without PsA may be driven by HLA-B amino acid position 45 ^27^. A study of 712 patients with PsA and 335 patients with psoriasis confirmed not to have arthritis by a rheumatologist demonstrated that the HLA alleles B*08, B*27, and B*38 are risk factors for the development of PsA, whereas HLA-C*06 is “protective” ^28^. HLA-B*27 was associated with early development of PsA among patients with psoriasis, whereas the presence of HLA-C*06 was associated with a delayed onset of PsA ^29^. HLA-B*27:05:02 is associated with increased risk of enthesitis, dactylitis, and symmetric sacroiliitis, whereas HLA-B*08:01:01 and HLA-C*07:01:01 haplotypes are associated with joint fusion and deformities, asymmetrical sacroiliitis, and dactylitis ^30^. Recent genome-wide association studies (GWAS) have identified SNPs near *HLA-C*, *TNFRSF9*, and *LCE3A* as more strongly associated with psoriasis than PsA, whereas SNPs near *IL-23R* and *TNFAIP3* were more strongly associated with PsA than PsC ^31^. Other genes identified as potential biomarkers for PsA are *NOTCH2NL*, *HAT1*, *CXCL10*, and *SETD2* ^32^.

***Soluble biomarkers.*** The markers CRP (hs-CRP), OPG, MMP-3, and the CPII:C2C ratio were found to distinguish patients with PsA from those with psoriasis without arthritis ^33^. In recent years, C-X-C motif chemokine 10 (CXCL10) was found to be a biomarker for the development of PsA in patients with psoriasis ^34^. Those who went on to develop PsA had higher serum levels of CXCL10 than those who did not. Additionally, serum CXCL10 dropped after PsA development. Upon examination of paired serum and synovial fluid samples from PsA patients, higher levels were seen in the synovial fluid in comparison to the blood; this indicates that CXCL10 may be a biomarker for the development of PsA in patients suffering from psoriasis and could be pathogenetically involved in its development ^35^. One group described that between cellular biomarkers, osteoclast precursor (OCP) was found in one-third of patients with psoriasis alone and in the majority of patients with PsA ^36^. An increase in OCP correlated with erosive disease. They also developed an antibody against a dendritic cell-specific transmembrane protein (DC-STAMP) which was associated with OCPs and could be an additional biomarker for identifying PsA early. These biomarkers are now being examined in psoriasis patients who go on to develop PsA ^37^.

With the advantages of all of these new biomarkers, we hope to have an earlier and accurate detection of these patients and treat them accordingly, but, since this is a multifactorial disease, it is likely that there will not be one biomarker but rather a combination of biomarkers.

## Pathogenesis of psoriatic arthritis

PsA pathogenesis involves multiple different factors, including genetic, immunologic, and environmental factors.

### Environmental factors

There is an association between upper respiratory airway streptococcal infection and guttate psoriasis. Vasey *et al*. found elevated levels of the Streptococcus exotoxin antibody anti-deoxyribonuclease B in PsA, but it was absent in patients with psoriasis alone ^38^.

Pattison *et al*. ^39^ compared the prevalence of environmental exposures among 98 British PsA and 163 psoriasis patients over a window of exposure that ranged from 5–10 years prior to the onset of arthritis. They identified physical trauma, rubella vaccination, oral ulcers, and moving to a new house as being associated with PsA. In 2011, Eder *et al*. ^40^ found in a case-control study that infections that required antibiotic treatment, injuries, and occupations that involved lifting heavy weights were associated with PsA, while there was an inverse association with smoking.

Among other factors, there is some evidence regarding obesity ^41^ and mechanical stress or trauma (Deep Koebner phenomenon). PsA patients have evidence of enthesophyte formation at mechanically exposed sites of the joint, while it is absent in healthy controls ^42^.

### Genetic factors

Psoriasis and PsA are associated with class I MHC alleles, mainly HLA-C*06, which is a major risk factor for psoriasis but not for PsA ^43^. HLA-B*27, HLA-B*38, HLA-B*08, and HLA-B*39 have been observed in PsA and associated with some PsA phenotypes ^44^.

The killer-cell immunoglobin-like receptor (KIR) genes were initially proposed in the early 2000s as genes conferring susceptibility to PsA ^45^. Later, Chandran *et al*. ^46^ proposed the activating KIR gene, explicitly *KIR2DS2*, as key in the susceptibility and the pathogenesis of PsA, since KIRs interplay with HLA-B Bw4 and HLA-C to augment the inflammatory response. These genes are coded on chromosome 19 but use the HLA-C molecules as ligands.

The endoplasmic reticulum aminopeptidase 1 (ERAP1) product is relevant to peptides binding to the MHC class I molecules, especially HLA-C*0602 and HLA-B*27 ^47^. SNPs related to genes relevant to immune function include loci containing genes involved in NF-kB signaling ( *REL*, *TNIP1*, *NFKBIA*, and *CARD14*), IFN signaling ( *IL28RA* and *TYK2*), T-cell regulation ( *RUNX3*, *IL13*, *TAGAP*, *ETS1*, and *MBD2*), and antiviral signaling ( *IFIH1*, *DDX58*, and *RNF114*) and genes involved in the IL-23 pathway that specifically implicate a role for T helper type 17 (Th17) cells ( *TNFAIP3*, *IL23R*, *IL12B*, *TRAF3IP2*, *IL23A*, and *STAT3*). Most of these have also been identified in PsA, but only two of these loci were independently identified in PsA, namely *IL12B* and *IL23R*, with the IL23R SNP being independent to the SNP found in psoriasis alone, and another region on chromosome 5q31 has also been identified as a marker for PsA ^48^.

### Immunological factors

T-cells are heavily involved in psoriasis and PsA. Activation of CD8 ^+^ T cells and natural killer (NK) cells in the psoriatic synovium and the disease’s response to therapeutic immunomodulation suggest that the immune system, particularly lymphocytes, has significant influence on PsA pathogenesis ^49^.

When Leijten *et al*. ^50^ compared PsA patients’ synovial fluid to rheumatoid arthritis synovial fluid, they saw that CD4 ^+^CD8 ^+^ lymphocytes were increased and that CD4 ^+^ Th17 and type 3 lymphocytes were also increased in the PsA population ^49^, the same cells that produce IL-17A and IL-22. There are different theories regarding the initial trigger of inflammatory response at multiple sites ^47, 51^.

In the skin, stressed keratinocytes release DNA that binds to the antibacterial peptide LL-37, and this stimulates plasmacytoid dendritic cells to release IFNα. This activates dermal dendritic cells, which will migrate to the draining lymph nodes and trigger T helper type 1 (Th1) and Th17 cells to differentiate. From here, Th1 and Th17 cells will migrate to the dermis and release IL-12, IL-17, IL-22, and TNFα, which promote keratinocyte proliferation.

In the gut, there is microbial dysbiosis that may trigger inflammation in the ileocolon and stimulate Th17 cells to release IL-23.

When there is trauma or biomechanical stress at the tendon insertion site, IL-23 is released, which activates Th17 cells and cytokines such as IL-22 and TNF, resulting in inflammation, bone erosion, and abnormal bone formation. IL-22 and other factors stimulate mesenchymal cells to differentiate into osteoblasts, forming enthesophytes in peripheral entheses and joints and syndesmophytes in the spine.

From nearby entheses or the bloodstream, Th17 cells, OCPs, and dendritic cells reach the joint. While here, OCPs differentiate into osteoclasts thanks to the increased expression of the receptor activator of NF-kB (RANK) ligand (RANKL) by the synoviocytes in the lining, combined with higher levels of TNF, IL-17, and RANKL expressed by infiltrating cells. All of this will lead to synovitis and bone resorption.

## Available therapy for psoriatic arthritis

Currently, there are about 17 targeted therapies considered for the management of active PsA; therefore, when assessing a patient, we should consider the major domains being affected (peripheral joints, axial disease, dactylitis, enthesitis, psoriasis, and nail disease) to make an informed decision regarding the pharmacologic therapy to be started. The goals of therapy are to achieve minimal disease activity, optimize functional status and quality of life, prevent structural damage, and avoid or minimize complications (from therapy and untreated disease) ^52^.

Conventional synthetic disease-modifying anti-rheumatic drugs (DMARDs) such as sulfasalazine, cyclosporine, and leflunomide have been shown to work for symptom relief with lower-grade evidence for methotrexate. None of these agents slow radiographic progression, help with axial symptoms, or relieve uveitis, enthesis, and dactylitis. When used, they can be considered for the treatment of peripheral arthritis ^53^.

Tumor necrosis factor inhibitors (TNFi) have been available for PsA patients since the 2000’s. These agents have demonstrated their effectiveness treating multiple domains of the disease, including peripheral and axial arthritis, enthesitis, dactylitis, skin psoriasis, and nail disease ^54^, and reducing radiographic progression. Available agents include etanercept, adalimumab, infliximab, golimumab, and certolizumab pegol ^55^.

In randomized controlled trials (RCTs), infliximab ^56^, golimumab ^57, 58^, and certolizumab ^59^ have been shown to be effective for enthesitis and dactylitis, whereas etanercept and adalimumab have been shown to control better enthesitis and dactylitis ^60, 61^. In 2017, the results of the phase III RAPID-PsA study established the efficacy of certolizumab in PsA patients in whom at least one DMARD previously failed or who previously received treatment with a TNFi ^59^. In two phase III trials, FUTURE 1 and 2, secukinumab, an IL-17A inhibitor, was tested. The first FUTURE-1 used 10 mg/kg secukinumab intravenously at weeks 0, 2, and 4, followed by subcutaneous secukinumab at a dose of either 150 mg or 75 mg every 4 weeks, or placebo ^63^. Primary outcome was American College of Rheumatology 20% (ACR20) response at 24 weeks. Significantly higher responses were observed for the two drug-treated groups compared to placebo. Secondary end points, including the ACR50 response and joint structural damage, were significantly better in the secukinumab groups than in the placebo group. FUTURE-2 included three doses: 75, 150, and 300 mg. While there were loading doses in this study, they were subcutaneous, not intravenous ^64^. The 75 mg dose did not work as well as the higher doses for the joints. The 300 mg was clearly better for the skin. Importantly, in both trials, secukinumab was effective for both TNFi-naïve and TNFi-experienced patients, although the 300 mg dose was more effective for the latter. FUTURE-5 ^65^ included 300 and 150 mg doses of secukinumab with a loading dose (LD) and 150 mg without loading dose as well as placebo, all using subcutaneous administration. All treatment groups did better than placebo, and there did not appear to be a difference between 150 mg with or without loading.

Another IL-17A inhibitor, ixekizumab, has been approved for PsA treatment. SPIRIT-P1 ^66^ included 417 TNF-naïve patients who were randomized to either subcutaneous ixekizumab 80 mg every 2 weeks or ixekizumab 80 mg every 4 weeks both following a loading dose of 160 mg, adalimumab 40 mg every other week, or placebo. Primary outcome was ACR20 at 24 weeks, which was achieved by 31% of the placebo-treated patients, 57% of the ixekizumab 80 mg every 4 weeks, 60% of those treated with ixekizumab every 2 weeks, and 51% of those treated with adalimumab. Ixekizumab improved HAQ scores, was effective for skin and nail disease, dactylitis, and enthesis, and was associated with less progression in radiologic damage ^67^. It was also effective in patients who had failed TNFi ^62^. In a new study presented in June 2019 at the EULAR congress, Mease *et al*. showed that ixekizumab was superior to adalimumab in treating PsA and plaque psoriasis in patients not previously exposed to b-DMARDs and who had an inadequate response to conventional DMARDs. A total of 36% of the ixekizumab group achieved PASI 100 and ACR50 versus 28% of the adalimumab group ( *P* <0.05). Composite treat-to-target outcomes, skin outcomes, enthesitis resolution, and quality of life related to skin were significantly better for the ixekizumab cohort.

Ustekinumab, an IL-12/-23 inhibitor, demonstrated efficacy in PsA patients in the phase III trials PSUMMIT 1 and 2. While its efficacy for arthritis is not quite as high as that of the anti-TNF agents, it works very well for psoriasis. It works for dactylitis and enthesitis as well ^69^.

Apremilast came out shortly after. It is a phosphodiesterase-4 inhibitor. In the PALACE 1, 2, and 3 trials, phase III studies, patients who were previously exposed to DMARDs or biologic agents and patients with no exposure (PALACE 4) were studied ^70– 72^ In the PALACE 3 trial, it demonstrated efficacy against placebo in patients who failed conventional DMARDs. The ACR20 response was good, although not as effective as the TNFi agents (ACR20 in 28% [dose of 20 mg twice daily] and 41% [30 mg twice daily] compared to placebo [18%] at week 16 and 56% [20 mg twice daily] and 63% [30 mg twice daily] at week 52). It was also effective for dactylitis and enthesitis. However, the effect on radiographic progression was not tested in these trials. In PALACE 4 **,** DMARD-naïve patients who received apremilast had an increased response to ACR20 (apremilast 20 mg twice daily 28%, apremilast 30 mg twice daily 30.7%, and placebo 15.9%) and ACR50, but not ACR70.

Abatacept, a CTLA4-Ig selective T-cell co-stimulation modulator, was proven effective in a phase III trial in PsA ^73^. In a study of 424 patients with PsA, half of whom received 125 mg of abatacept subcutaneously and the rest placebo, there was a significantly greater improvement with drug compared to placebo (ACR20 39% versus 22%), although the delta (difference between drug treated and placebo response) was lower than with the previously described therapies. The benefit was seen regardless of previous exposure to TNF inhibitors, and there was only modest impact on psoriasis lesions.

The Janus kinase (JAK) inhibitor tofacitinib, orally available and already approved for Rheumatoid Arthritis, has been tested in PsA. In OPAL BROADEN, a study of 422 TNFi-naïve patients, 107 received tofacitinib 5 mg twice daily, 104 received tofacitinib 10 mg twice daily, 106 received adalimumab 40 mg subcutaneously every other week, and 105 received placebo ^74^. The primary outcome was ACR20 response and change in HAQ at 12 weeks. ACR20 response was achieved by 50% of the 5 mg twice daily tofacitinib group and 61% of the 10 mg twice daily tofacitinib group, both statistically significantly different from the placebo group with 33% responders. In the comparator adalimumab-treated group, 52% achieved ACR20 at 12 weeks. HAQ scores were also reduced in all treatment groups compared to placebo. In OPAL BEYOND, 394 patients with inadequate response to TNFi were included. Of those, 131 received tofacitinib 5 mg twice daily, 132 received tofacitinib 10 mg twice daily, and 131 received placebo. ACR20 responses were achieved by 50% of those receiving tofacitinib 5 mg twice daily and 47% of those receiving 10 mg twice daily, compared with 24% of the placebo-treated patients ^75^. HAQ score reductions were significantly different from placebo with both doses of tofacitinib. There were no new safety signals in either of these two studies.

In early 2019, the results of the SEAM-PsA study were published. Mease *et al*. ^76^ examined the efficacy of methotrexate monotherapy compared to etanercept alone and the value of combining them both for the treatment of PsA. Patients with PsA were randomized to etanercept 50 mg subcutaneously once a week, methotrexate 20 mg orally once a week, or a combination of both etanercept and methotrexate. The results demonstrated that while methotrexate monotherapy resulted in a 50% ACR20 response *,* etanercept was superior to methotrexate. Moreover, the combination of etanercept and methotrexate was similar to etanercept alone, except for the skin responses, which were better with the combination. However, it should be noted that the dose of etanercept was 50 mg weekly rather than the psoriasis dose of 50 mg twice weekly.

## Therapies under investigation

Guselkumab is a human monoclonal antibody directed against the p19 subunit of IL-23; it is already approved for moderate-to-severe psoriasis treatment. In June 2019, the results from phase III trials were announced. DISCOVER 1 (n = 381) and DISCOVER 2 (n = 739) trials compared subcutaneous guselkumab to placebo over 52 and 100 weeks, respectively. The group stated that the medication met the primary end point (ACR20). Result of the trials will be presented soon ^77^. 

Risankizumab is a humanized immunoglobulin monoclonal antibody designed to selectively inhibit IL-23 by binding to its p19 subunit and was approved in April 2019 for the treatment of moderate-to-severe plaque psoriasis ^78^; there is an ongoing phase II trial in PsA ^79^.

Two JAK1-specific inhibitors, filgotinib ^80^ and upadacitinib, are currently under investigation. Table 2 documents the currently available therapies for PsA.

## Conclusions

PsA is a chronic inflammatory disease that comprises a clinical syndrome that could present with skin lesions, peripheral or axial arthritis, dactylitis, or nail lesions ^81^. Usually, PsA occurs after the development of psoriasis; therefore, screening these patients for the development of PsA is crucial so that they can be identified and treated early in order to decrease delay in consultation and its untoward effects. Despite the availability of multiple screening tools, we still need an algorithm to accurately identify patients early so they can have the benefit of therapy. Fortunately, in the past 10 years, the pathogenesis of PsA has been better understood, leading to several therapies, such as anti-TNFs, anti-IL-12/23, anti-IL-17, and anti-IL-23 agents, plus additional agents under investigation. Therefore, we anticipate that the treatment of PsA will become prompter and more aggressive so that joint damage is minimized. In addition, with the development of better therapies and more control over risk factors, PsA patients can experience fewer comorbidities as well as lowered mortality and improved quality of life and function.

**Table 2.** Summary of biologics agents investigated and/or approved in PsA.

Agent	Agent description	Study	Dose	Study size	Demographics	ACR20	PASI75
Certolizumab	Pegylated humanized anti-TNFα antigen binding fragment (Fab’) Binds soluble and membrane-bound TNFα	RAPID-PsA ^59^	Loading dose: 400 mg at week 0, 2, and 4, then either 200 mg SC every 2 weeks or 400 mg SC every 4 weeks VS P	409	**Mean age** 200 mg: 48.2 400 mg: 47.1 P: 47.3 **Female (%)** 200 mg: 53.6 400 mg: 54.1 P: 58.1 **Mean duration PsA (years)** 50 mg: 7.2 100 mg: 7.7 P: 7.6 **Prior use 1 DMARD (%)** 200 mg: 44.2 400 mg: 53.3 P: 54.4 **2 DMARDs** 200 mg: 52.9 400 mg: 44.5 P: 44.1 **Prior TNF exposure (%)** 200 mg: 22.5 400 mg: 17.0 P: 19.1	**Week 12** 200 mg: 58% 400 mg: 51.9% P: 24.3% **Week 24** 200 mg: 63.8% 400 mg: 56.3% P: 23.5%	**Week 12** 200 mg: 46.7% 400 mg: 47.4% P: 14% **Week 24** 200 mg: 62.2% 400 mg: 60.5% P: 15.1%
**Adalimumab**	Human monoclonal Ab. Binds soluble and membrane-bound TNFα	ADEPT ^60^	A 40 mg SC every 2 weeks VS P	315	**Mean age** A: 48.6 P: 49.2 **Male (%)** A: 56 P: 55 **Mean duration PsA (years)** A: 9.8 P: 9.2	**Week 12** A: 58% P: 14%	**Week 12** A: 49% **Week 24** A: 59% P: 1%
**Ixekizumab**	IL17 inhibitor	SPIRIT-P1 ^66^	TNF-naïve patients 80 mg SC every 2 weeks 80 mg SC every 4 weeks following a loading dose of 160 mg, A 40 mg EOW, or P	417	**Mean age** Every 4 weeks: 49.2 Every 2 weeks: 49.8 P: 50.6 A 40 mg every 2 weeks: 48.6 **Male (%)** Every 4 weeks: 42.1 Every 2 weeks: 46.6 P: 45.3 A 40 every 2 weeks: 50.5 **Mean duration PsA (years)** Every 4 weeks: 13.8 Every 2 weeks: 14 P: 13.8 A 40 mg every 2 weeks: 12.7	**24 weeks** 80 mg every 2 weeks: 62.1% 80 mg every 4 weeks: 57.4% A: 57.9% P: 30.2%	**24 weeks** Every 4 weeks: 71.2% Every 2 weeks: 79.7% P: 10.4% A 40 mg every 2 weeks: 54.4%
**Apremilast**	Phosphodiesterase-4 inhibitor	PALACE 3 ^72^	20 mg twice daily 30 mg twice daily VS P	505	**Female (%)** 20 mg: 53 30 mg: 53 P: 54 **Mean age** 20 mg: 49.5 30 mg: 49.9 P: 49.5 **Mean duration PsA (years)** 20 mg twice daily: 7.7 30 mg twice daily: 7.5 P: 6.8	**Week 16** 20 mg twice daily: 28% 30 mg twice daily: 42% P: 18% **Week 52** 20 mg twice daily: 56% 30 mg twice daily: 63%	**Week 16** 20 mg twice daily: 20% 30 mg twice daily: 21% P: 8% **Week 52** 20 mg twice daily: 29% 30 mg twice daily: 39%
**Abatacept**	CTLA4Ig inhibitor	ASTRAEA trial ^73^	ABA SC 125 mg /week VS P	424 60% prior TNFi	**Mean age** ABA: 51 P: 49.8 **Female (%)** ABA: 56.8 P: 53.1 **Mean duration PsA (years)** ABA: 8.3 P: 8.8	**24 weeks** ABA: 39.4% P: 22.3%	**24 weeks** ABA: 16.4% P: 10.1%
**Tofacitinib**	Inhibitor JAK3-1	OPAL BROADEN ^74^	Tofacitinib at a 5 mg dose by mouth twice daily Tofacitinib 10 mg dose by mouth twice daily A at a 40 mg dose SC once every 2 weeks P with a blinded switch to 5 mg tofacitinib dose at 3 months or P with a blinded switch to the 10 mg tofacitinib dose at 3 months	394	**Mean age** 5 mg: 49.4 10 mg: 46.9 P: 47.7 A: 47.4 **Female (%)** 5 mg: 53 10 mg: 60 P: 53 A: 47 **Mean duration PsA (years)** 5 mg: 7.3 10 mg: 5.4 P: 6.4 A: 5.3	**12 weeks** 5 mg: 50% 10 mg: 61% P: 33% A: 52%	**12 weeks** 5 mg: 43% 10 mg: 44% P: 15% A: 39%
**Adalimumab**		GENOVESE 2007 ^85^	A 40 mg EOW VS P Followed by open label study: A 40 mg EOW	100	**Mean age** A: 50.a P: 47.7 **Male (%)** A: 57 P: 51 **Mean duration PsA (years)** A: 7.5 P: 7.2	**Week 12** A: 39% P: 16%	
**Etanercept**	Fusion protein. Extracellular binding portion of TNF receptor (p75) dimerized on human IgG1	Mease 2000 ^86^	E 25 mg SC twice week VS P	60	**Mean age** E: 46 P: 43.5 **Male (%)** E: 53 P: 60 **Mean duration PsA (years)** E: 9.0 P: 9.5	**12 weeks** E: 73% P: 13%	**12 weeks** E: 26% P: 0%
		Mease 2004 ^87^	E 25 mg SC twice weekly × 24 weeks VS P	205	**Mean age** E: 47.8 P: 47.3 **Male (%)** E: 57 P: 45 **Mean duration** **PsA (years)** E: 9.0 P: 9.2	E: 59% P: 15% Sustained at 24 weeks	E: 23% P: 3%
		Extension study 48 weeks ^88^				E: 64% P: 50%	
**Infliximab**	Mouse-human chimeric anti TNF monoclonal Ab. Binds soluble and membrane-bound TNFα	IMPACT ^89^	I 5 mg/kg IV at weeks 0, 2, 6, and 14 VS P	104	**Mean age** I: 45.7 P: 45.2 **Male (%)** I: 58 P: 58 **Mean duration PsA** ( **years)** I: 16.9 P: 19.4	**Week 16** I: 67,3% P: 11.5 %	**Week 16** I: 68% P: 0%
		IMPACT 2 ^56^	I IV 5 mg/kg at weeks 0, 2, 6, 14, and 22 VS P	200	**Mean age** I: 47.1 P: 46.5 **Male (%)** I: 71 P: 51 **Mean duration PsA (years)** I: 8.4 P: 7.5	**Week 12** I: 58% P: 11%	I: 64% P: 2%
**Golimumab**	Human monoclonal Ab. Binds soluble and membrane-bound TNFα	GO-REVEAL ^90,^	50 mg or 100 mg SC	405	**Mean age** 50 mg: 45.7 100 mg: 48.2 P: 47.0 **Male (%)** 50 mg: 89 100 mg: 86 P: 69 **Mean duration PsA (years)** 50 mg: 7.2 100 mg: 7.7 P: 7.6	**Week 14** 50 mg: 51% 100 mg: 45% P: 9%	**Week 14** 50 mg: 40% 100 mg: 58% P: 2.5%
**Ustekinumab**	Human mAb IgG1. Binds to p40 of IL-12 and IL-23	PSUMMIT 1 ^91^	Previous inadequate response to MTX 45 mg 90 mg P Week 0, 4, and every 12 weeks	615	**Mean age** 45 mg: 48 90 mg: 47 P: 48 **Male (%)** 45 mg: 51.7 90 mg: 56.9 P: 52.4 **Mean duration PsA (years)** 45 mg: 3.4 90 mg: 4.9 P: 3.6	**Week 24** 45 mg: 42.2% 90 mg: 49.5% P: 22.8%	**Week 24** 45 mg: 57.2% 90 mg: 62.4% P: 11%
		PSUMMIT 2 ^92^	45 mg 90 mg P Week 0, 4, and every 12 weeks VS P with cross over to UST 45 mg at week 24, 28, and 40	312	**Female (%)** 45 mg: 53.4 90 mg: 53.3 P: 51 **Mean age** 45 mg: 49 90 mg: 48 P: 48 **Mean duration PsA (years)** 45 mg: 5.3 90 mg: 4.5 P: 5.5	45 mg: 43.7% 90 mg: 43.8% P: 20.2% **Anti-TNF exposure** 45 mg: 36.7% 90 mg: 34.5% P: 14.5%	45 mg: 51.3% 90 mg: 55.6% P: 5% **Anti-TNF exposure** 45 mg: 45.5 % 90 mg: 48.8% P: 2.0%
Apremilast		PALACE 4 ^93^ DMARD-naive	20 mg/twice daily 30 mg/twice daily VS P At week 16 or 24, P patients were rerandomized to apremilast	527	**Mean age** 20 mg: 49.2 30 mg: 48.4 P: 50.5 **Female (%)** 20 mg: 54.3 30 mg: 54.5 P: 48.9 **Mean duration PsA (years)** 20 mg: 15.3 30 mg: 15.4 P: 16.8	**Week 16** 20 mg: 28% 30 mg: 30.7% P: 15.9 **%** **Week 52** 20 mg: 53.4% 30 mg: 58.7%	**Week 16** 20 mg: 17.3% 30 mg: 25.7% P: 10.8% **Week 52** 20 mg: 41% 30 mg: 31.9%
**Secukinumab**	IL-17 inhibitor	FUTURE 2 - ^94^	Sec SC 300 mg 150 mg 75 mg VS P Week 1, 2, 34, and every 4 weeks after	397		Sec75: 50.3% Sec150: 64.4% Sec300: 69.4%	Sec75: 58.4% Sec150: 73.3% Sec300: 79.5%
		FUTURE 5 ^65^	Sec 300 mg or 150 mg with loading dose, 150 mg without loading dose, or P. All groups received Sec or P at baseline, weeks 1, 2, and 3 and then every 4 weeks from week 4	996	**Mean age** 300 mg + loading dose: 48.9 150 mg + loading dose: 48.4 150 mg: 48.8 P: 49 **Female (%)** 300 mg + loading dose: 51.4 150 mg + loading dose: 49.5 150 mg: 4.5.9 P: 51.5 **Mean duration PsA (years)** 300 mg + loading dose: 48.9 150 mg + loading dose: 48.4 150 mg: 48.8 P: 49	**Week 16** 300 mg with loading dose: 62.6% 150 mg with loading dose: 55.5% 150 mg without loading dose: 59.5% P: 27.4%	**Week 16** 300 mg + loading dose: 70% 150 mg + loading dose: 60 150 mg: 58.1 P: 12.3

ABA, Abatacept; A, adalimumab; E, etanercept; EOW, every other week; I, infliximab; IV, intravenous; mAb, monoclonal antibody; mg, milligrams; P, placebo; PsA, psoriatic arthritis; SC, subcutaneous; Sec, secukinumab; TNFi, tumour necrosis factor inhibitor; VS, versus
